# The effects of XMRV gene expression on the mouse prostate

**DOI:** 10.1186/1742-4690-8-S1-A223

**Published:** 2011-06-06

**Authors:** Daniel Rauch, Sirosh Bokhari, John Harding, Lee Ratner

**Affiliations:** 1 Division of Molecular Oncology, St Louis, MO, 63110, USA

## 

Xenotropic murine leukemia virus related virus (XMRV) has been the subject of intense investigation since its discovery and initial characterization in human prostate carcinoma. Notwithstanding the important and ongoing controversies surrounding detection methods, seroprevalence, disease association, or viral origin, we sought to determine whether XMRV gene expression was sufficient to promote prostate pathology in a transgenic mouse model. Using the probasin promoter to drive prostate specific expression of XMRV genes in a transgenic mouse (PRO-XMRV) in vivo, we compared prostates of age matched male littermates over time. Using immunohistochemistry to stain envelope proteins we have been able to detect XMRV gene expression in the prostate of 3 of 3, 11-15 month old mice with the highest levels found in the lateral lobes. The lateral lobes also exhibit thickened layers of glandular epithelia that contain elevated numbers of bromodeoxyuridine retaining cells. While this model produces wild-type particles that can be detected in the urine by RT-PCR, infection is not thought to be occurring due to the absence of the receptor, XPR-1, in mouse cells. To determine if viral infection and integration is required for XMRV-related prostate pathology, we have also created a transgenic strain in which XPR-1 is expressed in the mouse prostate (PRO-XPR1). Breeding PRO-XMRV mice with PRO-XPR1 mice will allow us to test whether XMRV integration or gene expression can cause more advanced prostate pathology in vivo. With these XMRV mouse models we seek to address the question that remains unanswered to date as to whether XMRV is capable of causing prostate dysplasia or cancer in vivo.

